# Editorial Comment: Does previous standard percutaneous nephrolithotomy impair retrograde intrarenal surgery outcomes?

**DOI:** 10.1590/S1677-5538.IBJU.2021.0253.1

**Published:** 2021-10-01

**Authors:** Marco Antonio Fortes

**Affiliations:** 1 Hospital Quinta D´Or Instituto D`OR de Ensino e Pesquisa Departamento de Urologia Minimamente Invasiva Rio de JaneiroRJ Brasil Departamento de Urologia Minimamente Invasiva do Instituto D`OR de Ensino e Pesquisa - Hospital Quinta D´Or, Rio de Janeiro, RJ, Brasil

## COMMENT

Assintomatic infundibula stricture is a late complication of percutaneous nephrolithotomy (PCNL) and can have serious consequences. Parsons et al (1) in previous assessment of imaging exams found a rate of 2.3% of this pathology and the mean time to stenosis detection was 9 months (range 2-24).

The rigid nephroscopy can usually reach the renal pelvis when the puncture is made in the lower pole but reaching upper pole calyces and interpolar calyces without placing undue torque on the renal parenchyma can be challenging, especially in obese patients with low-lying kidneys because of hindrance from the iliac crest. In these cases a steeper torque associated with a large expansion orifice may result in the formation of scar tissue and stricture.

This prospective study investigated the impact of PCNL on retrograde intra-renal surgery (RIS) outcomes found a rate of infundibula stricture much higher than those previously described, reaching a quarter of operated patients, probably due to the factors described above (2).

We conclude that, in addition to previous imaging exams, the surgeon must be prepared to find and treat the infundibula stricture in patients previously submitted to PCNL.
